# The hypotensive effect of acute and chronic AMP-activated protein kinase activation in normal and hyperlipidemic mice

**DOI:** 10.1016/j.vph.2015.07.010

**Published:** 2015-11

**Authors:** Fiona H. Greig, Marie-Ann Ewart, Eilidh McNaughton, Josephine Cooney, Corinne M. Spickett, Simon Kennedy

**Affiliations:** a Institute of Cardiovascular and Medical Sciences, College of Medical, Veterinary & Life Sciences, University of Glasgow, Glasgow G12 8QQ, UK; b School of Life & Health Sciences, Aston University, Birmingham B4 7ET, UK

**Keywords:** Mean arterial blood pressure, Hypotension, AMPK, AICAR, Hyperlipidemia

## Abstract

AMP-activated protein kinase (AMPK) is present in the arterial wall and is activated in response to cellular stressors that raise AMP relative to ADP/ATP. Activation of AMPK in vivo lowers blood pressure but the influence of hyperlipidemia on this response has not been studied. ApoE^−/−^ mice on high fat diet for 6 weeks and age-matched controls were treated with the AMPK activator, AICAR daily for two weeks. Under anesthesia, the carotid artery was cannulated for blood pressure measurements. Aortic tissue was removed for in vitro functional experiments and AMPK activity was measured in artery homogenates by Western blotting. ApoE^−/−^ mice had significantly raised mean arterial pressure; chronic AICAR treatment normalized this but had no effect in normolipidemic mice, whereas acute administration of AICAR lowered mean arterial pressure in both groups. Chronic AICAR treatment increased phosphorylation of AMPK and its downstream target acetyl-CoA carboxylase in normolipidemic but not ApoE^−/−^ mice. In aortic rings, AMPK activation induced vasodilation and an anticontractile effect, which was attenuated in ApoE^−/−^ mice. This study demonstrates that hyperlipidemia dysregulates the AMPK pathway in the arterial wall but this effect can be reversed by AMPK activation, possibly through improving vessel compliance.

## Graphical abstract

**Figure:**
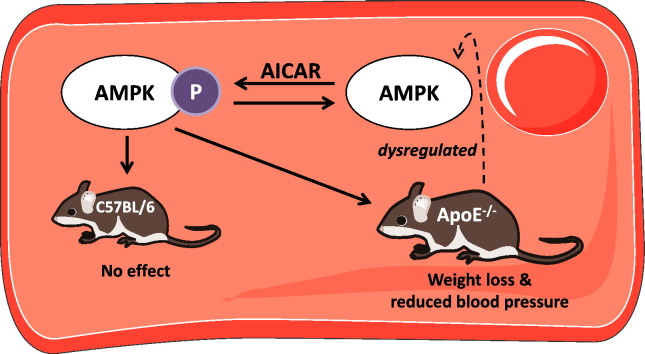

## 1 Introduction

AMP-activated protein kinase (AMPK) is an enzyme with a central role in cellular energy homeostasis that is activated in response to a change in the cellular balance of AMP to ADP/ATP. Often described as the cell's fuel gauge, it becomes activated in response to cellular stressors including inflammation, hypoxia and oxidant stress [1]. Recent evidence (reviewed in [2]) points to a vasculoprotective role for AMPK activation that may be mediated through inducing endothelial NO production [3], [4], preventing EC-monocyte adhesion [5] and positively regulating vascular redox balance via upregulating expression of manganese superoxide dismutase [6], and reducing reactive oxygen species generation in response to hyperglycemia [7]. AMPK can also reduce the proliferative effects of stimuli such as platelet derived growth factor and angiotensin II (Ang-II) [8], and is likely to be intimately involved in vascular remodeling [9].

AMPK is a trimer of α (catalytic) and β and γ (regulatory) subunits which, although ubiquitous, has tissue-specific subunit isoform expression. In vascular cells, isoforms containing the α_1_ subunit predominate [10] while α_2_ predominates in cardiac tissue [11]. AMPK activation involves phosphorylation of Thr^172^ on the α subunit by upstream AMPK kinases (AMPKK), primarily LKB-1 [12] and CaMKKβ [13]. Activated AMPK phosphorylates several downstream targets, including acetyl-coenzyme A carboxylase (ACC) [14]. At a cellular level, this stimulates fatty acid oxidation, mitochondrial biogenesis and glucose uptake, inhibition of fatty acid synthesis, cholesterol production and gluconeogenesis [2]. In atherosclerosis, the presence of oxidized low density lipoproteins increases endoplasmic reticulum (ER) stress [15] and causes a 40-fold increase in expression of protein phosphatase 2A (PP2a), the enzyme responsible for inactivating AMPK [16]. Recent evidence suggests that activation of AMPK in atherosclerosis has beneficial effects including reversing ER stress [15] and stimulating reverse cholesterol transport from foam cells to reduce plaque area in mice deficient in apolipoprotein E (ApoE^−/−^) [17], [18].

Hypertension is a risk factor in development of atherosclerosis and dysfunction of the endothelium may be a feature common to both pathologies [19]. The ApoE^−/−^ mouse develops spontaneous hypercholesterolemia and atherosclerosis with the first signs of disease occurring at 6 to 8 weeks with features accelerated by high fat feeding [20], [21]. Some studies [22] have measured an increased blood pressure in ApoE^−/−^ mice while others suggest no difference from control, non-atherosclerotic mice [23], [24]. Previous studies have shown that acute administration of the AMPK activating agent, 5-aminoimidazole-4-carboxamide-1-β-d-ribofuranoside (AICAR) reduces mean arterial blood pressure (MAP) in both rodents and humans [25], [26]. Furthermore, spontaneously hypertensive rats dosed with AICAR showed an acute drop in MAP that was not seen in control WKY rats, suggesting that AMPK could play a role in reducing hypertension [27]. Long-term administration of AICAR or resveratrol, another activator of AMPK, also reduced blood pressure in obese Zucker rats [28], [29] and Ang-II-induced hypertensive mice [30]. In vitro experiments using aortae from mice lacking AMPKα1 indicate that AMPK may improve endothelial function via endothelial nitric oxide synthase (eNOS) phosphorylation [31], while chronic activation of AMPK in mice reversed the deleterious effects of the vasoconstrictor 20-HETE on eNOS [32]. Collectively, these studies suggest that activation of AMPK may reduce blood pressure though an effect on vascular eNOS. However, what is not clear is how hyperlipidemia or established fibrofatty plaques affect AMPK activity within the arterial tree and if this attenuates the ability of AMPK activation to modulate blood pressure. Consequently, the aims of this study were to assess the effect of high fat feeding on MAP in ApoE^−/−^ mice and whether chronic activation of AMPK in vivo affects blood pressure and vascular AMPK activity. A further aim was to study how the presence of atherosclerotic lesions affects the hypotensive response to acute AMPK activation and the vasodilator response to AMPK activating agents in vitro.

## 2 Methods

### 2.1 Animal models

All in vivo experiments were performed in accordance with the United Kingdom Animals (Scientific Procedure) Act of 1986. Mice were housed at the University of Glasgow and maintained on 12 hour cycles of light and dark and at ambient temperature. Two strains of mice were used: ApoE^−/−^ (bred in-house) and the genetic background control (C57BL/6, Harlan). In all experiments, age-matched male mice were used. C57BL/6 mice were fed a standard chow diet while ApoE^−/−^ mice commenced a high fat diet (21% lard and 0.15% cholesterol, SDS) at 8 weeks of age. High fat diet was continued for either six weeks to induce hyperlipidemia without arterial lesions or for 12 weeks, a time point at which we have previously demonstrated that atherosclerotic lesions are present in the arterial tree [33]. At the end of the experimental protocol, mice were used to study the effects of acute or chronic AMPK activation on blood pressure and vascular function as outlined below. For some of the vascular function experiments, mice deficient in the main vascular isoform of AMPK, α1 (AMPKα^−/−^) and their wild-type littermates (S129 strain) were used. These were originally obtained from Professor Benoit Viollet (Institut Cochin, Paris, France), bred in-house and age-matched to the 12 week fat-fed ApoE^−/−^ mice.

### 2.2 In vivo hemodynamic measurements

To study the effect of chronic AMPK activation on blood pressure and the effect of hyperlipidemia, ApoE^−/−^ mice (on diet for 4 weeks) were administered daily i.p. injections of the AMPK activating agent, AICAR (Toronto Research Chemicals Inc.) or an equivalent volume of vehicle (distilled water) at a dose of 400 mg/kg [34], [35]. An age-matched group of C57BL/6 mice was also treated concurrently with either AICAR or vehicle. Weight was monitored throughout to allow AICAR dose adjustment if required. For all groups, treatment continued for 14 days at which time hemodynamic measurements were performed under terminal anesthesia. At the end of the procedure, blood was removed by cardiac puncture into EDTA-containing Vacutainers for measurement of plasma myeloperoxidase (MPO), spleen, heart and liver were also removed and weighed. The carotid artery and aorta were dissected out, cleaned of surrounding fat and connective tissue and snap-frozen in liquid nitrogen for later analysis. To study the effect of acute AICAR administration on blood pressure in C57BL/6 mice and ApoE^−/−^ mice with established atherosclerosis following 12 weeks of high fat diet, mice were injected with a single dose of AICAR (400 mg/kg i.p.) or vehicle 45 min before induction of terminal anesthesia and recording of blood pressure.

Mice were anesthetized using an isoflurane mixture and a polyurethane cannula (Harvard Apparatus) filled with heparinized saline was inserted into the carotid artery. The cannula was connected to an Elcomatic E751A pressure transducer and MP35 data acquisition system (BIOPAC Systems Inc.). The recordings were made using commercially available software and data was acquired for at least 10 min in each animal. MAP was derived from analysis of at least 5 points within the 10 minute analysis period.

### 2.3 Western blotting

Aortae were pulverized in liquid nitrogen and re-suspended in ice-cold cell lysis buffer (50 mM Tris pH 7.4, 50 mM NaF, 1 mM Na_4_PPi, 1 mM EGTA, 1 mM EDTA, 1% Triton X-100, 1 mM DTT and 1% cocktail of protease inhibitors). All the samples (10 μg) were run on NuPAGE Novex 4–12% Bis-Tris mini gels (Life Technologies), transferred to nitrocellulose membrane and analyzed with the following primary antibodies: AMPKα (1:1000; Cell Signaling Technology), phospho-AMPKα (1:1000; Cell Signaling Technology), ACC (1:1000; Cell Signaling Technology), phospho-ACC (1:1000; Cell Signaling Technology), GAPDH (1:40000; Abcam), and α tubulin (1:5000; Abcam). Protein bands were visualized using an enhanced chemiluminescence detection kit (Thermo Scientific) and the density was quantified using a GS-800™ Calibrated Densitometer (BioRad) and Quantity One BioRad software.

### 2.4 Measurement of MPO and plasma lipids

The MPO content of plasma from C57BL/6 and ApoE^−/−^ mice was analyzed using a mouse MPO ELISA kit (Hycult® Biotech Inc.). Plasma samples were diluted 1 in 16 in dilution buffer and the assay was performed as per the manufacturer's instructions. Absorbance was measured spectrophotometrically at 450 nm using a SpectraMax M2 microplate reader. Plasma total cholesterol, HDL cholesterol and triglycerides were measured in undiluted plasma on an ILAB 600 clinical chemistry analyzer, using Roche kits for HDL cholesterol. Triglyceride and cholesterol kits were from Randox Laboratories. All values were calibrated using the assigned kit calibrators and checked against the relevant quality controls.

### 2.5 Small vessel wire myography

To assess the effects of AMPK activation on vascular tone in atherosclerotic mice and mice treated with AICAR, mouse aorta was removed, cleaned of all fat and connective tissue and cut into 2 mm rings. In most experiments the endothelium was removed by gently rubbing the interior of the vessel with a human hair and removal confirmed by lack of (< 10%) vasodilator response to 10^− 6^ M acetylcholine. Artery rings were mounted on two stainless steel wires in a four channel wire myograph (Danish Myo Technology), set to an optimum tension of 9.8 mN [36] and allowed to equilibrate for at least 30 min before use. Vessels were bathed in Krebs–Henseleit buffer (118 mM NaCl, 4.7 mM KCl, 1.2 mM MgSO_4_, 25 mM NaHCO_3_, 1.03 mM KH_2_PO_4_, 11 mM glucose and 2.5 mM CaCl_2_) at 37 °C and gassed continuously with 95% O_2_ and 5% CO_2_. Vessel viability was checked with 40 mM KCl and preconstriction was produced by addition of a submaximal concentration (3 × 10^− 8^ M) of the thromboxane A_2_ mimetic, U46619. Cumulative concentration–response curves to AICAR (10^− 4^ M–10^− 2^ M) or AMPK activator A769662 (10^− 6^ M–5 × 10^− 4^ M) were performed with addition of drug at 10 minute intervals. For all experiments, data were expressed as a percentage of relaxation of the U46619-induced tone. We also measured the effect of preincubation of AMPK activating agents on the constrictor response to U46619. In this case, 2 mM AICAR or 30 μM A769662 was added 45 min prior to U46619 contraction.

### 2.6 Statistical analysis

All results are presented as mean ± SEM where n represents the number of mice used for each experiment. In vivo data were analyzed using a two-way ANOVA followed by Bonferroni's post hoc test. Myography data were analyzed using two-way ANOVA to compare the complete concentration–response curve. Biochemical data and plasma lipids were compared by one-way ANOVA for multiple comparisons. In all cases a p value of < 0.05 was taken to indicate statistical significance.

## 3 Results

### 3.1 Blood pressure and heart rate

**Fig. 1:**
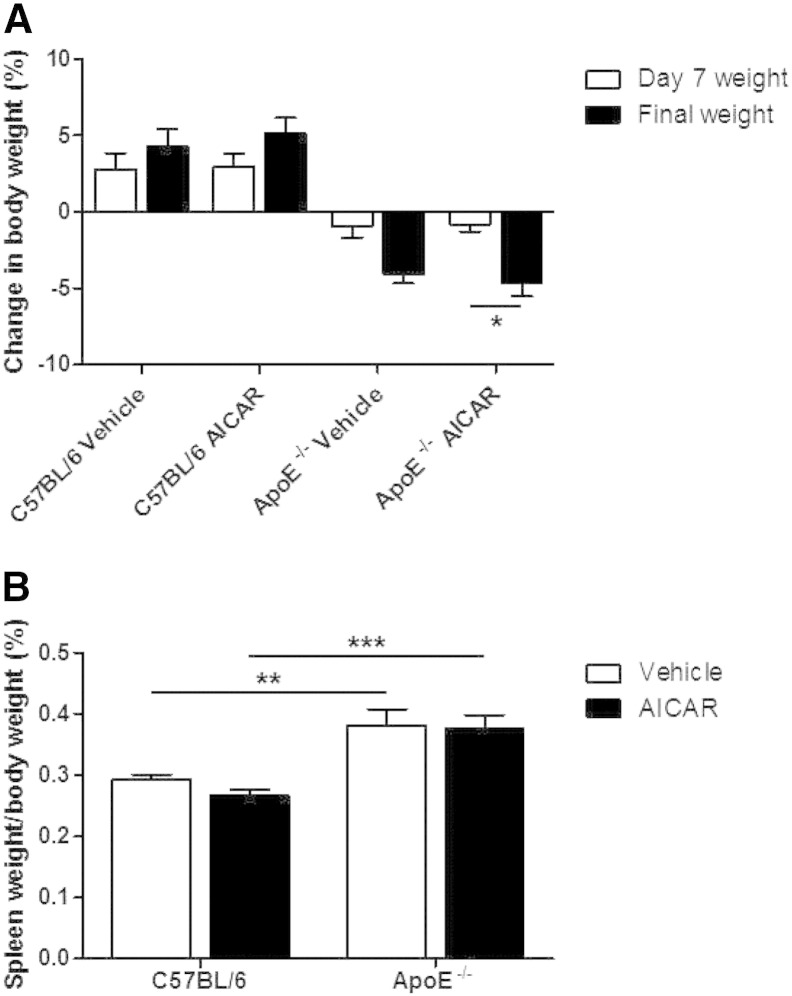
Body and organ weight fluctuations in experimental mice. (A) The weight of each mouse was recorded at the beginning, midpoint (day 7) and at the end of the procedure and from these the percentage change was calculated. *p < 0.05; n = 9–10. (B) Spleen weights were calculated as a percentage of the final body weight of the mice. **p < 0.01, ***p < 0.001; n = 9–10.

In the chronic AICAR experimental groups, the ApoE^−/−^ mice were fed high fat diet for 4 weeks before commencing either vehicle or AICAR daily for two weeks (a total duration of 6 weeks of high fat feeding). This resulted in an increased body weight compared to age-matched C57BL/6 mice (25.0 ± 0.45 g vs. 28.0 ± 0.6 g; p < 0.001; n = 9–10). C57BL/6 mice gained weight during two week treatment with either vehicle or AICAR. In contrast both groups of ApoE^−/−^ mice lost weight with a significantly greater percentage weight loss relative to starting weight in the AICAR treatment group (Fig. 1A). Organ weights were also measured following 2 weeks of AICAR administration. Heart weight did not differ between strains or with AICAR administration. Liver weight was reduced in ApoE^−/−^ mice compared to C57BL/6 mice and administration of AICAR increased liver weights in both strains of mice compared to saline-treated controls (Table 1.). There was also a marked increase in spleen weight in both ApoE^−/−^ groups that was unaffected by AICAR treatment (Fig. 1B). The group of ApoE^−/−^ mice which were maintained on high fat diet for 12 weeks to study acute effects of AICAR on blood pressure also had a significantly increased body weight compared to age-matched C57BL/6 mice (26.75 ± 0.66 g vs. 30.23 ± 0.88 g; p < 0.01; n = 9), which was accompanied by an enlarged spleen but no difference in heart weight (Table 1).

**Fig. 2:**
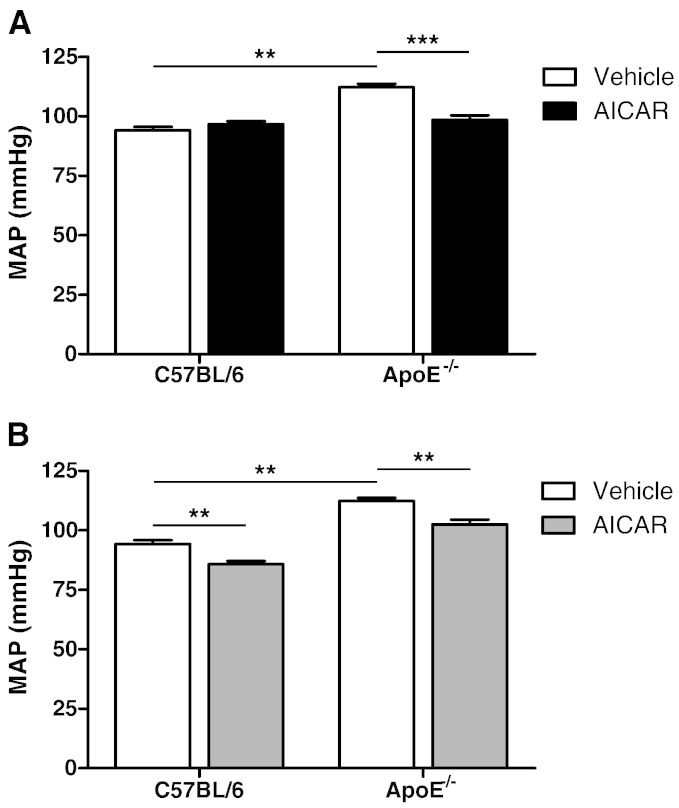
Mean arterial pressure (MAP) of C57BL/6 and ApoE^−/−^ mice treated chronically or acutely with AICAR. MAP was measured by cannulation of the left common carotid artery. (A) ApoE^−/−^ mice were fed high fat diet for a total of 6 weeks with vehicle or AICAR injected daily for the final 2 weeks. C57BL/6 mice received chow diet and either vehicle or AICAR. **p < 0.01, ***p < 0.001; n = 6–10. (B) MAP of ApoE^−/−^ mice on high fat diet for 12 weeks and age-matched C57BL/6 controls administrated vehicle or AICAR 45 min before blood pressure measurement. **p < 0.01; n = 6–10.

In ApoE^−/−^ mice on high fat diet for 6 weeks, MAP was significantly increased compared to age-matched controls. Two weeks of chronic AICAR treatment had no effect on MAP in control mice but significantly lowered the value in ApoE^−/−^ mice (Fig. 2A). Similar effects on diastolic pressure were seen in ApoE^−/−^ mice (Table 1) while systolic values, which were significantly elevated in ApoE^−/−^ mice, were not lowered by AICAR administration. Heart rate was also increased in vehicle-treated ApoE^−/−^ mice compared to C57BL/6 controls (Table 1). AICAR administration had no effect on heart rate in either strain of mouse. ApoE^−/−^ mice fed the high fat diet for 12 weeks also had a significantly raised MAP, DAP, SAP and HR values, which were reduced in mice injected with AICAR 45 prior to blood pressure measurement (Table 2). However, in contrast to chronic administration of AICAR, a single, acute administration of AICAR also significantly reduced MAP in the control mice (Fig. 2B and Table 2).

### 3.2 Expression of AMPK and ACC

The expression of phosphorylated and total AMPK and its downstream target, ACC, were investigated in both mouse aortae and liver to assess the influence of hyperlipidemia and chronic AMPK activation with AICAR on this pathway. These mice had therefore received a total of 6 weeks of high fat diet and 2 weeks of AICAR or vehicle treatment.

**Fig. 3:**
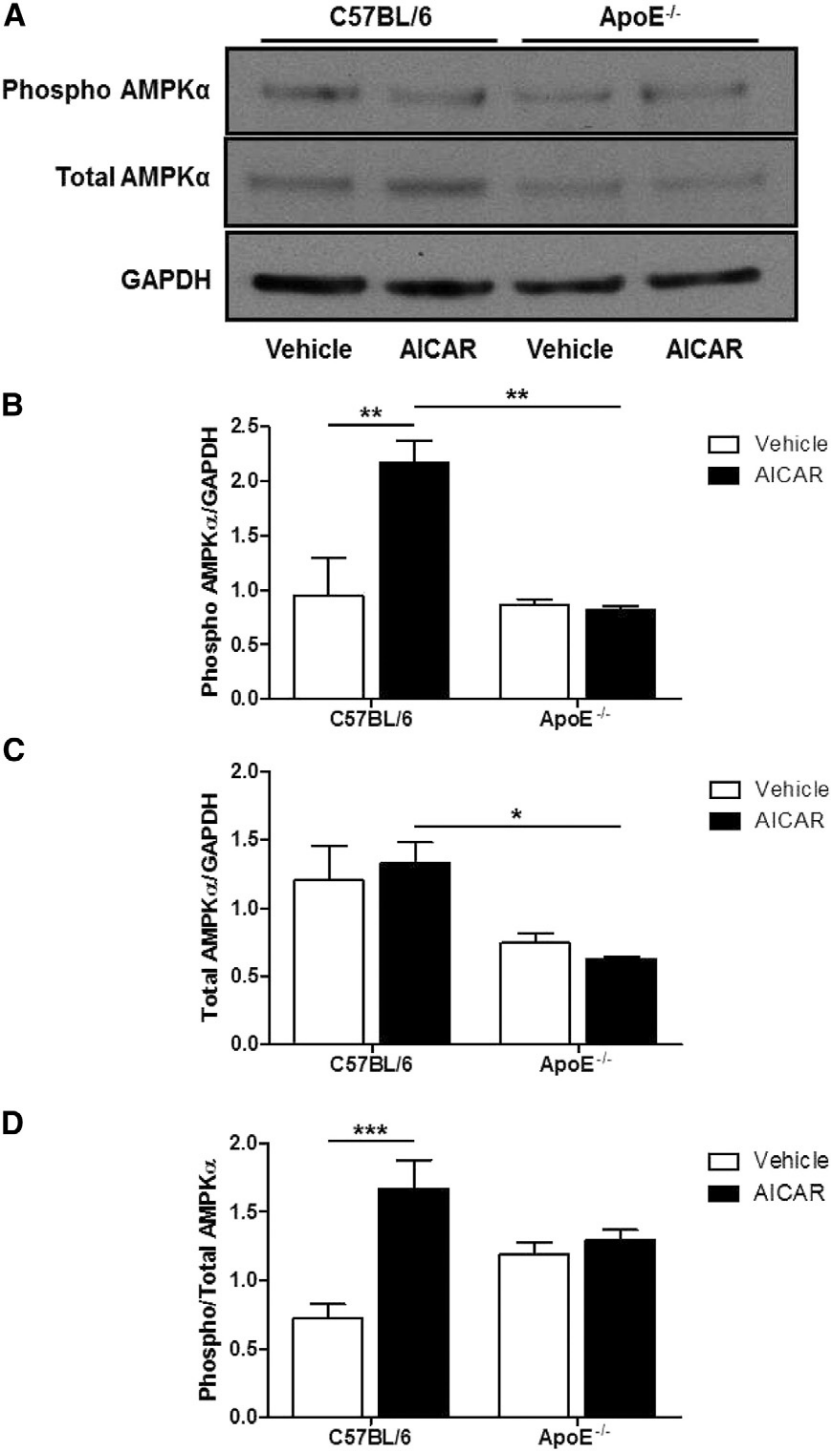
AMPKα expression in aortae of C57BL/6 and ApoE^−/−^ mice treated with AICAR. (A) Blots shown are representative. (B) Phosphorylated and (C) total AMPKα were divided by GAPDH to adjust for protein loading to measure the amount of each form of the enzyme. (D) A ratio of phosphorylated to total AMPKα was calculated to measure the phosphorylation status of the enzyme. *p < 0.05, **p < 0.01, ***p < 0.001; n = 4.

Chronic treatment of control mice with AICAR did not result in any significant increase in the expression of total AMPK in the aorta; however, the expression of phosphorylated AMPK did increase (Fig. 3A and B). In ApoE^−/−^ mice, there was a reduction in both total AMPK and phosphorylated AMPK expression compared to C57BL/6 mice and chronic AICAR treatment did not increase the phosphorylation of AMPK in these mice. Consequently, the ratio of phosphorylated to total AMPK was significantly increased in control mice treated with AICAR, while no effect was seen in ApoE^−/−^ mice (Fig. 3D).

**Fig. 4:**
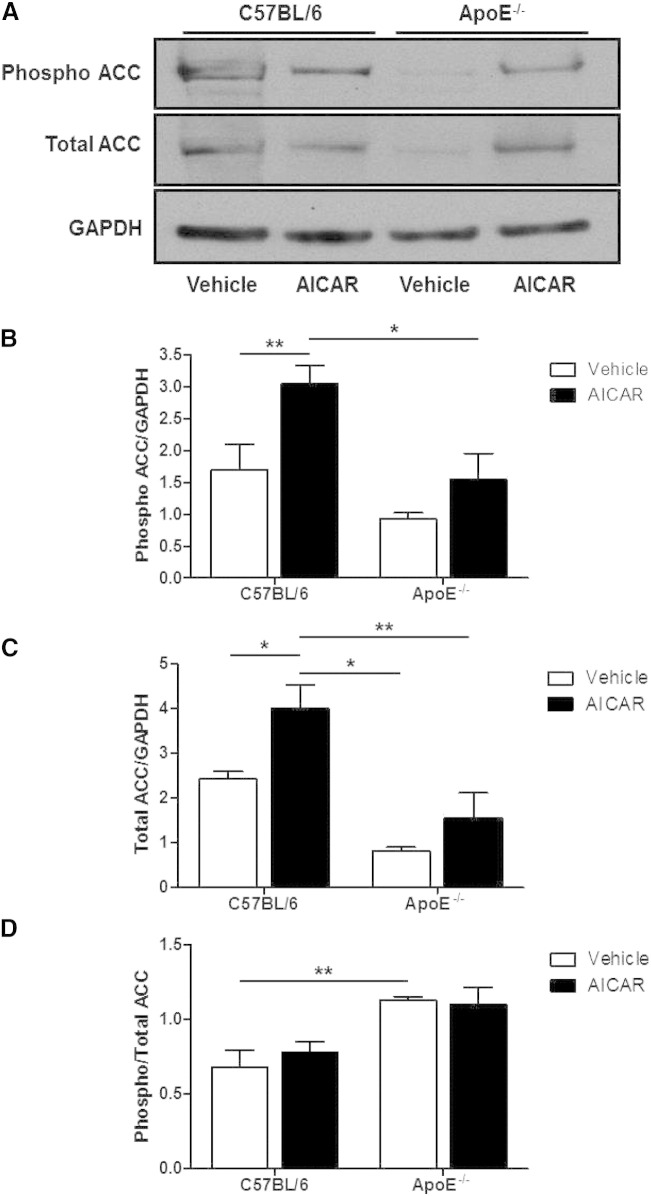
ACC expression in aortae of C57BL/6 and ApoE^−/−^ mice treated with AICAR. (A) Blots shown are representative. (B) Phosphorylated and (C) total ACC were divided by GAPDH to adjust for protein loading to measure the amount of each form of the enzyme. (D) A ratio of phosphorylated to total ACC was calculated to measure the phosphorylation status of the enzyme. *p < 0.05, **p < 0.01; n = 4.

In control mice, total ACC expression in the aorta was significantly increased by chronic AICAR treatment and an increase in phosphorylated ACC was also observed (Fig. 4). In vehicle-treated ApoE^−/−^ mice, total ACC expression in the aorta was significantly lower compared to control mice but AICAR treatment raised the expression of both total and phosphorylated ACC expression (Fig. 4). Consequently, the ratio of phosphorylated to total ACC in the aorta did not change in control or ApoE^−/−^ mice as a result of AICAR treatment. However, this ratio was significantly lower in C57BL/6 mice compared to ApoE^−/−^ mice (Fig. 4).

In homogenized samples of liver from vehicle-treated animals, total AMPK expression was significantly reduced in the ApoE^−/−^ mouse (Table 3). Chronic AICAR treatment had no effect on total AMPK expression in either strain and did not increase AMPK phosphorylation in either strain. The ratio of phosphorylated to total AMPK was higher in ApoE^−/−^ mice and was found to be significantly reduced by chronic AICAR treatment (Table 3). There was no significant difference in total ACC expression in C57BL/6 and ApoE^−/−^ liver homogenates after vehicle treatment. Chronic AICAR administration had no effect on total ACC in C57BL/6 mice but significantly increased it in ApoE^−/−^ mice. Phosphorylated ACC levels in the liver were significantly lower in ApoE^−/−^ compared to C57BL/6 mice but chronic AICAR treatment had no effect on phosphorylated ACC in either strain. The ratio of phosphorylated to total ACC in liver was not significantly different between strains and AICAR had no effect in either strain (Table 3).

### 3.3 Plasma MPO and lipids

The influence of chronic AICAR treatment on the MPO content of plasma from control mice and ApoE^−/−^ mice fed on high fat diet for 6 weeks was investigated. ApoE^−/−^ mice dosed with vehicle had a dramatic increase in MPO levels compared to vehicle-treated C57BL/6 mice (151.9 ± 17.9 ng/ml vs. 598.2 ± 55.0 ng/ml; p < 0.001; n = 8–9). AICAR treatment had no effect on MPO levels in control mice but significantly increased MPO in the plasma of ApoE^−/−^ mice (598.2 ± 55.0 ng/ml vs. 762.7 ± 71.12 ng/ml; p < 0.05; n = 9). Compared to age-matched C57BL/6 mice, ApoE^−/−^ mice on high fat diet for 6 weeks had significantly raised total cholesterol (15.52 ± 0.75 mg/dl vs. 1.99 ± 0.11 mg/dl; p < 0.05, n = 9–10) and triglycerides (0.91 ± 0.16 mg/dl vs. 0.32 ± 0.04 mg/dl; p < 0.05, n = 9–10) while HDL-cholesterol was not raised (2.15 ± 0.12 mg/dl vs. 1.80 ± 0.10 mg/dl; n = 9–10). Chronic treatment with AICAR had no effect on the lipid profile in C57BL/6 mice (data not shown) but significantly raised HDL-cholesterol (2.15 ± 0.12 mg/dl vs. 3.62 ± 0.26 mg/dl; p < 0.05, n = 9–10) and total cholesterol (15.52 ± 0.75 mg/dl vs. 19.43 ± 0.99 mg/dl; p < 0.05, n = 9–10) in the ApoE^−/−^ mouse without any effect on triglycerides (0.91 ± 0.16 mg/dl vs. 0.98 ± 0.11 mg/dl; n = 9–10).

### 3.4 Vascular function in vitro

**Fig. 5:**
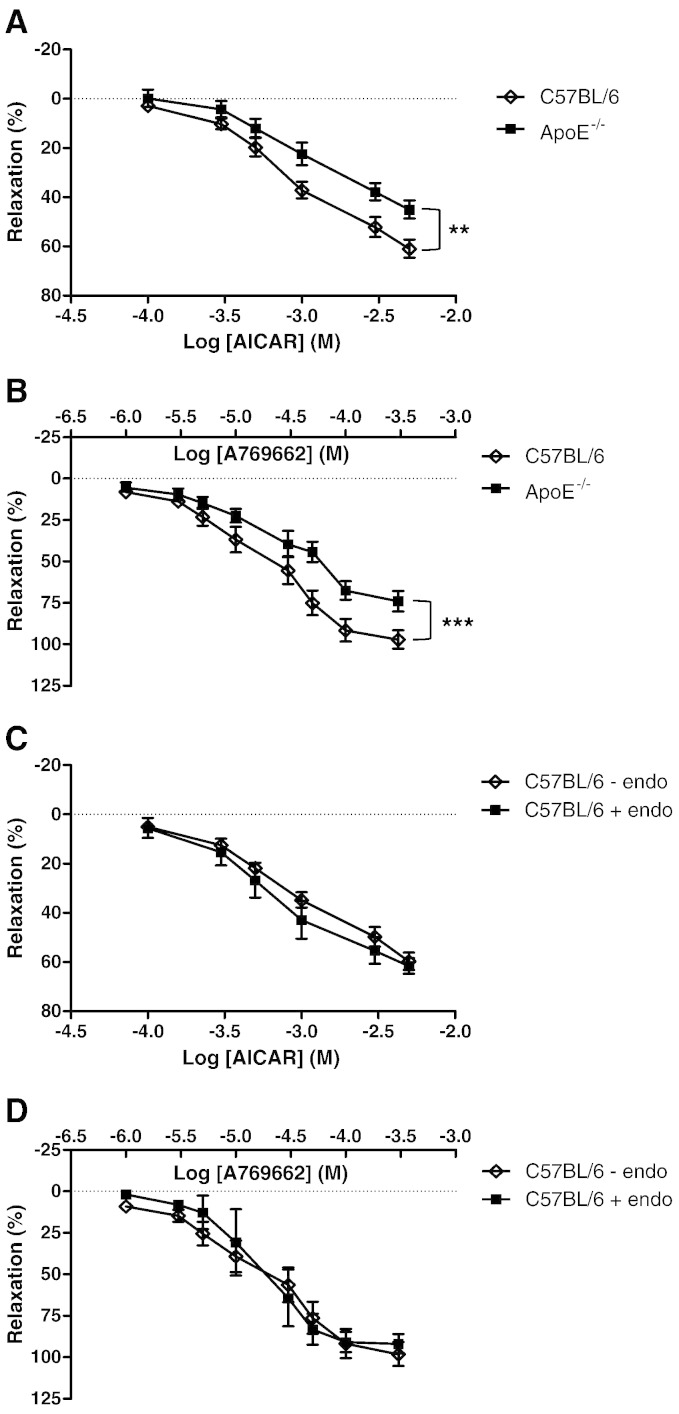
Relaxation of C57BL/6 and ApoE^−/−^ aortic rings to AMPK activating agents. (A) Denuded aortic rings from 3 month high fat-fed ApoE^−/−^ mice and age-matched C57BL/6 controls were precontracted with U46619 and concentration–response curves to (A) AICAR and (B) A769662 were produced using small vessel wire myography. In endothelium-intact aortic rings from C57BL/6 mice, AICAR (C) and A769662 (D) also induced a relaxation of similar magnitude to denuded rings. **p < 0.01, ***p < 0.001; n = 7.

In these experiments, aortae from 12 week fat-fed mice were used as we have shown previously that the arteries contain fatty lesions [33]. In denuded aortic rings, both AMPK activating agents AICAR and A769662 produced a relaxation which was significantly reduced in 12 week fat-fed ApoE^−/−^ mice (Fig. 5A and B). In endothelium-intact rings from C57BL/6 mice, relaxation of a similar magnitude to denuded rings (Fig. 5C and D) was observed. In 12 week fat-fed ApoE^−/−^ mice, the presence of intact endothelium (defined as a 50% relaxation in response to acetylcholine) could not be confirmed (n = 7).

**Fig. 6:**
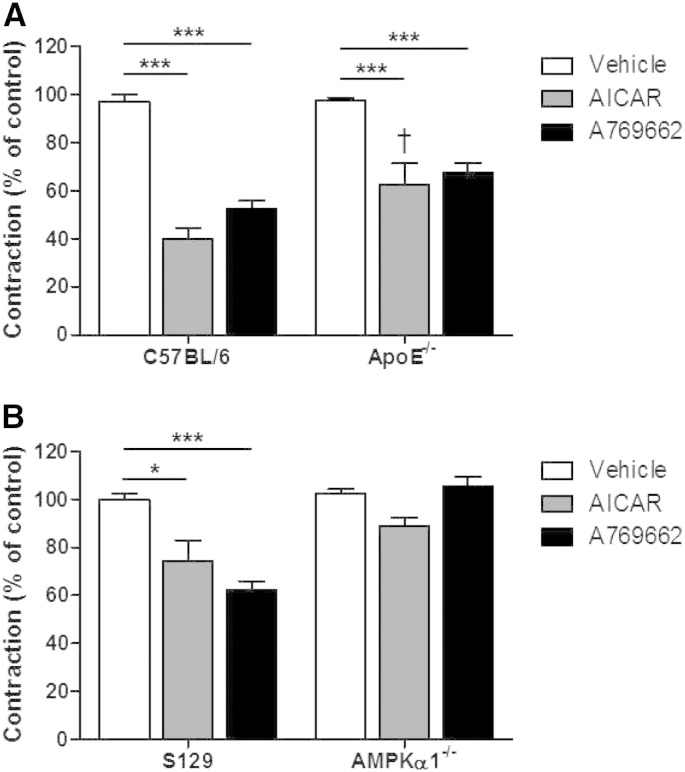
Effect of preincubation of AMPK activating agents on the constrictor response to U46619 in mouse aortae. (A) 2 mM AICAR or 30 μM A769662 were added for 45 min prior to U46619 contraction in 3 month fat-fed ApoE^−/−^ mice and aged-matched C57BL/6 mice. ***p < 0.001; ^†^p < 0.05 vs. C57BL/6 AICAR; n = 4–9. (B) 2 mM AICAR or 30 μM A769662 were added for 45 min prior to U46619 contraction in AMPKα1^−/−^ mice and their wild-type littermates (S129). *p < 0.05, ***p < 0.001; n = 4–6.

Since activation of AMPK can induce an anticontractile effect, we tested the effect of a single addition of either AICAR (2 mM) or A769662 (30 μM) prior to contracting the ring with U46619. Both agents produced a significant anticontractile effect but this was significantly reduced with AICAR in the ApoE^−/−^ aorta (Fig. 6). To investigate this further we repeated the experiments using AMPKα1^−/−^ or wild-type littermates (S129). In the KO animals, the anticontractile effect of both AICAR and A769662 was lost, confirming that it is due to activation of AMPK (Fig. 6).

## 4 Discussion

In this study we demonstrate for first time that chronic activation of AMPK can normalize the raised MAP in hyperlipidemic ApoE^−/−^ mice. Pulse pressure was much lower in ApoE^−/−^ mice and was dramatically increased by chronic AMPK activation, so we speculated that the ApoE^−/−^ mouse has reduced vascular compliance that underlies the raised MAP. In support of this, ApoE^−/−^ mice fed a high fat diet for 3 months, a time point at which atherosclerotic lesions are evident [33], had a reduced vasodilator response to AMPK activating agents and were more sensitive to contractile agents. Since ApoE^−/−^ mice also had reductions in the amount of phosphorylated and total AMPKα and ACC following AICAR treatment, we conclude that the AMPK pathway is dysregulated in the artery wall of hyperlipidemic mice and this may accelerate may of the processes which contribute to atherosclerotic lesion formation.

We sought to study the response to chronic AMPK activation in ApoE^−/−^ mice at a time point where atherosclerotic lesions are not established, since there is little information on blood pressure and vascular function early in the disease process. Mean arterial, diastolic and systolic pressures as well as heart rate were all elevated while pulse pressure was reduced in ApoE^−/−^ mice fed on the high fat diet for 6 weeks compared to C57BL/6 controls. Some previous studies [37] reported no differences in hemodynamic measurements between age-matched 6 week old ApoE^−/−^ and C57BL/6 mice, but these mice were not fed on a high fat diet, which would be expected to accelerate pathological changes in vessel structure and function. However, other studies in young ApoE^−/−^ mice have shown elevated systolic and diastolic pressures and heart rates compared to C57BL/6 control mice [22], and a decrease in arterial elasticity caused by excessive extracellular matrix deposition may be responsible [38]. ApoE^−/−^ mice maintained on the same diet for 3 months had a similarly raised MAP compared to age-matched C57BL/6 mice, indicating that the hypertension is established early after fat feeding in this model and does not worsen as atherosclerotic lesions are established. Inflammatory burden was also raised even after only 6 weeks fat feeding, with enlarged spleens [39] and raised plasma MPO in ApoE^−/−^ mice compared to controls.

Chronic AMPK activation by AICAR normalized MAP in ApoE^−/−^ mice without affecting heart rate, whereas acute administration had a hypotensive effect in both atherosclerotic ApoE^−/−^ mice and age-matched C57BL/6 mice. AICAR has previously been shown to have a hypotensive effect when given acutely to spontaneously hypertensive rats and humans [25], [26], [27] while chronic AMPK activation lowered blood pressure in insulin resistant rats [28], [29] and in Ang-II-induced hypertension [30]. The acute reduction in blood pressure may be due to increased nitric oxide bioavailability from the vascular endothelium [27]. The hypotensive response to acute AICAR in our study was the same in C57BL/6 and ApoE^−/−^ mice, suggesting a fully functional endothelium following 12 weeks of fat feeding. However, this is not supported by previous reports showing very early dysfunction in this model [40]. The hypotensive effect may be mediated by a direct effect on the vascular smooth muscle cells which also express AMPK and indeed, we present data showing relaxation to two AMPK activating agents in endothelium denuded aortic rings in this study (Fig. 5).

Interestingly, chronic AICAR administration also caused significant weight loss in the ApoE^−/−^ mouse only. Previous studies have reported that hypothalamic AMPK activity is involved in modifying food intake and activation of the AMPK pathway induces hyperphagia and weight gain [41], [42]. However, a recent study demonstrated no change in body weight in rats chronically dosed with AICAR for up to 8 weeks but did show increased leptin sensitivity, reduced adiposity and increased oxidative capacity of white adipose tissue [43]. In control mice, weight gain was the same in AICAR and vehicle-treated animals, but significant weight loss was seen in ApoE^−/−^ mice treated with AICAR. In the study by [43], chronic dosing with AICAR induced up to 10% reduction in food intake. We did not monitor food intake in the present study, but the normal weight gain in the C57 mice treated with either vehicle or AICAR suggests food intake was unaffected by AICAR. The weight loss in the ApoE^−/−^ group may have been due to dysregulated AMPK signaling caused by hyperlipidemia, a feature observed in previous studied with hypertensive rats [27] and insulin resistant rats [28]. However we cannot rule out a reduction in food intake in the ApoE^−/−^ mice. Dysregulation of AMPK and its downstream target ACC in hyperlipidemic mice is supported by our data on vascular expression and phosphorylation of these proteins. Not unexpectedly, chronic AICAR treatment upregulated the phosphorylation status of AMPK and its downstream target ACC in the aorta of C57BL/6 mice, which is likely to indicate an increase in activity of the enzyme. In complete contrast, ApoE^−/−^ mice had significantly reduced expression of phosphorylated and total AMPKα and ACC in response to AICAR. Vehicle-treated ApoE^−/−^ mice also had reduced levels of total ACC compared to vehicle-treated C57BL/6 mice, which resulted in an elevation in the phosphorylated to total ratio of ACC. The reduced expression and function of AMPK may be a result of the developing atherosclerosis but would certainly render the animal less sensitive to changes in cellular redox balance and perhaps less able to deal with metabolic disturbances. The reduced AMPK expression may be due to higher levels of protein phosphatase 2A, which is partly responsible for inactivating AMPK [16].

In the liver, there was a reduction in the total amount of AMPKα in ApoE^−/−^ mice and AICAR did not increase phosphorylation. Phosphorylated ACC was also reduced in ApoE^−/−^ mice. In a previous investigation, AMPK activity was increased 3.6-fold in the liver only 1 h after administration of AICAR [28], which suggests the rapid metabolism of AICAR by the liver and subsequent activation of AMPK signaling. In the ApoE^−/−^ mouse, the reduction in total AMPK could have affected the ability of AICAR to induce phosphorylation. The chronic nature of the AICAR dosing may also have resulted in desensitization of the target protein. A reduction in total hepatic AMPK has been shown in other disease models such as obese Zucker rats [29]. Since AMPK activation would increase hepatic fatty acid oxidation and therefore the catabolism of free fatty acids and triglycerides [44], a reduction in the activity of the enzyme or its ability to be activated by agents such as AICAR could adversely affect lipid profile and exacerbate diet-induced hyperlipidemia.

As anticipated, even after only 6 weeks of high fat diet ApoE^−/−^ mice displayed features of generalized inflammation such as increased spleen weight and raised plasma MPO, the latter being consistent with published reports [45], [46], [47]. The plasma MPO content found in the present study is much higher than those found previously in mice deficient in low density lipoprotein receptor fed on a high fat diet for 6 weeks (160 ng/ml) [48] but this could be due to gender or strain differences. In addition, AICAR treatment significantly increased MPO in the plasma of ApoE^−/−^ mice but not normolipidemic control mice. This result was unexpected as a previous study has shown AMPK activation to cause a decrease in MPO levels, albeit in lung tissue and not in the circulation [49]. A possible explanation is activation of AMPK in neutrophils by AICAR leading to the release of MPO [50]. ApoE^−/−^ mice may have increased circulating neutrophils or perhaps increased neutrophil AMPK content which could account for the lack of effect of AICAR on MPO levels in C57BL/6 mice, though we did not measure neutrophil counts in this study so this remains speculative.

Previous reports indicate that AMPK activation may induce vasodilation via activation of eNOS and in this study we demonstrated that a single, acute administration of AICAR lowered blood pressure in both C57BL/6 and ApoE^−/−^ mice. Therefore, we performed some experiments in isolated mouse aortic rings from C57BL/6 and 3 month fat-fed ApoE^−/−^ mice to investigate how AMPK activation affects vascular tone. We found that both AICAR and A769662 induced vasodilation that did not require an intact endothelium. To avoid the effect of endothelial dysfunction in the ApoE^−/−^ group, we studied only denuded aortic rings. The vascular effects of both agents were due to AMPK activation since they were absent in aortic rings from global AMPKα1^−/−^ mice (Fig. 6) and it is likely that AMPK present in the medial smooth muscle is responsible for the vasodilator effect. The blunted response to both AMPK activators in the ApoE^−/−^ mouse and the reduction in the anticontractile effect in response to AMPK activation is in line with the AMPK and ACC expression data, which showed altered expression and function following high fat diet in the ApoE^−/−^ mouse. An anticontractile effect in response to AMPK activation at the level of the endothelium has been published [32] but our data showing that the anticontractile effect in denuded vessels is attenuated in atherosclerotic animals has not been reported previously.

Here we demonstrate that the elevated blood pressure in 6-week fat-fed ApoE^−/−^ mice can be normalized without any change in heart rate by chronic activation of AMPK. The beneficial effect of chronic activation may be due to improvements in arterial compliance. The ApoE^−/−^ mice also had dysregulated AMPK expression and phosphorylation in the artery wall, which may contribute to disease progression. In ApoE^−/−^ mice with established fibrofatty plaques, AICAR could still induce a hypotensive response when administered acutely, suggesting that even although the AMPK pathway is dysregulated, sufficient activity remains to dilate blood vessels. In vitro experiments with denuded aortic rings showed that AMPK activity in the vascular smooth muscle has anticontractile and vasodilator activity which could maintain the hypotensive response in the ApoE^−/−^ mouse when endothelial function is compromised. Based on our findings, the role of AMPK in maintaining vascular health and blood pressure deserves further investigation, together with the therapeutic effects of AMPK activators.

## Figures and Tables

**Table 1:** Weight and hemodynamic measurements of C57BL/6 and ApoE^−/−^ mice after 14 days of AICAR administration.

	C57BL/6 mice (6 weeks age matched)		ApoE ^−/−^ mice (6 weeks high fat diet)	
Vehicle	AICAR	Vehicle	AICAR
Start weight (g)	25.0 ± 0.5	21.7 ± 0.4⁎⁎⁎	28.0 ± 0.6⁎⁎⁎	26.5 ± 0.6‡‡‡
Final weight (g)	26.1 ± 0.3	22.9 ± 0.4⁎⁎⁎	26.9 ± 0.6	25.3 ± 0.5†, ‡‡
Heart weight (%)	0.54 ± 0.02	0.50 ± 0.01	0.51 ± 0.02	0.47 ± 0.01
Liver weight (%)	4.91 ± 0.13	5.55 ± 0.18⁎⁎	4.42 ± 0.16⁎	5.24 ± 0.10†††
Spleen weight (%)	0.29 ± 0.01	0.27 ± 0.01	0.38 ± 0.03⁎⁎	0.38 ± 0.02‡‡‡
MAP (mm Hg)	94.1 ± 1.5	96.8 ± 1.2	112.2 ± 1.5⁎⁎⁎	98.5 ± 1.8
DAP (mm Hg)	86.8 ± 1.8	88.5 ± 1.0	110.0 ± 1.7⁎⁎⁎	88.4 ± 1.8
SAP (mm Hg)	105.1 ± 1.3	110.3 ± 2.2	115.3 ± 1.6⁎⁎⁎	112.8 ± 2.2
PP (mm Hg)	18.3 ± 0.9	21.8 ± 2.6	5.3 ± 0.5⁎⁎⁎	24.4 ± 1.5†††
HR (bpm)	317.7 ± 13.9	344.7 ± 20.2	410.7 ± 15.1⁎⁎	381.9 ± 15.1

Start weight refers to weight on commencing vehicle or AICAR treatment. Organ weight was taken as a percentage of the final body weight. MAP, heart rate (HR), diastolic arterial pressure (DAP), systolic arterial pressure (SAP) and pulse pressure (PP) were calculated from the blood pressure trace. n = 6–10.

⁎ p < 0.05.

⁎⁎ p < 0.01.

⁎⁎⁎ p < 0.001 vs. C57BL/6 vehicle.

† p < 0.05.

††† p < 0.001 vs. ApoE^−/−^ vehicle.

‡‡ p < 0.01.

‡‡‡ p < 0.001 vs. C57BL/6 AICAR.

**Table 2:** Weight and hemodynamic measurements after acute AICAR administration in C57BL/6 and 12 week fat-fed ApoE^−/−^ mice.

	C57BL/6 mice (12 weeks age matched)		ApoE ^−/−^ mice (12 weeks high fat diet)	
Vehicle	AICAR	Vehicle	AICAR
Body weight (g)	26.7 ± 0.7		30.2 ± 0.8⁎⁎	
Heart weight (%)	0.69 ± 0.04		0.67 ± 0.06	
Liver weight (%)	5.42 ± 0.11		4.09 ± 0.2⁎⁎⁎	
Spleen weight (%)	0.27 ± 0.02		0.55 ± 0.07⁎⁎	
MAP (mm Hg)	94.3 ± 1.6	83.2 ± 1.8⁎	112.3 ± 3.5⁎⁎⁎	102.3 ± 2.9††
DAP (mm Hg)	79.2 ± 2.3	64.1 ± 1.9⁎⁎	107.4 ± 2.1⁎⁎⁎	98.0 ± 1.1†
SAP (mm Hg)	109.5 ± 2.5	103.9 ± 3.09	117.8 ± 2.3⁎	106.3 ± 3.7†
PP (mm Hg)	31.0 ± 0.8	39.6 ± 2.2	10.4 ± 3.0	8.3 ± 2.6
HR (bpm)	440.1 ± 7.5	400.4 ± 21.2⁎	486.7 ± 16.7⁎	350.1 ± 30†

Organ weight was taken as a percentage of the body weight. MAP, HR, DAP, SAP and PP were calculated from the blood pressure trace. n = 6–10.

⁎ p < 0.05.

⁎⁎ p < 0.01.

⁎⁎⁎ p < 0.001 vs. C57BL/6 vehicle.

† p < 0.05.

†† p < 0.01 vs. ApoE^−/−^ vehicle.

**Table 3:** Expression of AMPK and ACC in homogenized liver samples from C57BL/6 and ApoE^−/−^ mice after 14 days of AICAR administration.

	C57BL/6 mice		ApoE ^−/−^ mice	
Vehicle	AICAR	Vehicle	AICAR
Phospho AMPK	0.54 ± 0.05	0.46 ± 0.03	0.70 ± 0.07	0.58 ± 0.09
Total AMPK	1.01 ± 0.09	0.94 ± 0.14	0.42 ± 0.02⁎⁎	0.46 ± 0.02‡‡
Phosphorylated/total AMPK	0.53 ± 0.02	0.51 ± 0.07	1.68 ± 0.18⁎⁎⁎	1.28 ± 0.05†, ‡‡‡
Phospho ACC	2.07 ± 0.18	1.84 ± 0.27	1.31 ± 0.07⁎	1.24 ± 0.23
Total ACC	1.58 ± 0.18	1.79 ± 0.27	0.98 ± 0.06	1.03 ± 0.15^‡^
Phosphorylated/total ACC	1.34 ± 0.13	1.03 ± 0.08	1.34 ± 0.07	1.20 ± 0.06

Values for total ACC and phosphorylated ACC are given as a ratio of the density of the band corresponding to the protein of interest relative to the density of the GADPH band. A ratio of phosphorylated to total ACC was used to assess activation of the enzyme. n = 4.

⁎ p < 0.05.

⁎⁎ p < 0.01.

⁎⁎⁎ p < 0.001 vs. C57BL/6 vehicle.

† p < 0.05 vs. ApoE^−/−^ vehicle.

‡‡ p < 0.01.

‡‡‡ p < 0.001 vs. C57BL/6 AICAR.
